# Supplementary material for: Characterization of a High-Affinity Copper Transporter CTR1a in the White-Nose Syndrome Causing Fungal Pathogen Pseudogymnoascus destructans
Source: J Fungi (Basel). 2024 Oct 21;10(10):729. doi: 10.3390/jof10100729 (PMC11509074; doi:10.3390/jof10100729)
Supplement: Supplementary file 1 [file jof-10-00729-s001.zip › Supplemental File S1 - CTR Multiple sequence alignment.pdf]

Supplemental File S1. BLAST sequence alignment of Cu-transport homologs from *S. cerevisiae (Sc)*, *C. neoformans (Cn)*, and *P. destructans (Pd).* Protein sequences were retrieved using the identifiers YPR124W (*Sc*CTR1), YHR175W(*Sc*CTR2), CNAG_00979(*Cn*CTR2), CNAG_01872 (*Cn*CTR2), CNAG_07701(*Cn*CTR1), VC83_00191 (*Pd*CTR1a), and VC83_04814(*Pd*CTR1b). Large portions of non-homologous alignment sequence are designated by a bold "X" with the subscript number corresponding to the number of un-aligned amino acids.

```
ScCTR1          MEGMNMGSSMNMDAMSSASKTVASSMASMSMDAMSSASKTILSSMSSMSMEAMSSASKTL    60
PdCTR1a         ------------------------------------------------------MADNPF    6
CnCTR1          ------------------------------------------------------------    0
CnCTR2          ------------------------------------------------------------    0
CnCTR3/4        ------------------------------------------------------------    0
PdCTR1b         ------------------------------------------------------------    0
ScCTR2          ------------------------------MDDKKT-----------WSTVTLRTFNQLV    19

ScCTR1          ASTMSSMASMSMGSSSMSGMSMSMSSTPTSSASAQTTSDSSMSGMSGMSSSDNSSSSGMD    120
PdCTR1a         A------TSTPTSGDDMSGMDM-------------------SHGSSHG---SS-----HG    33
CnCTR1          --------------MDMSGMD----------------------GMDHM---SS-----TS    16
CnCTR2          -----------------------MNHGD-----------HSKHTMPDM---D--------    15
CnCTR3/4        --------------MDMGNMGMGMGMGM-----------DSGHNHSHM---NMGSGHGAD    32
PdCTR1b         -------------------------MSH-----------SMGHG-------DHD-----A    12
ScCTR2          T------SSLIGYSKKMDS--MNHKMEG-----------NAGHDHSDM---HMG-----D    52

ScCTR1          MDMSMGMNYYLTPTYKNYPVLFHHLHANNSGKAFGIFLLFVVAAFVYKLLLFVSWCLEVH    180
PdCTR1a         SSSGMSMVMTFQNNP-STPLFSTAWTPTGTGSYAGTCIFLIVFAVLFRVLLALKARQEAR    92
CnCTR1          SNMSMSMKMYFHGTIGGDLLWFASWMPSSAGATVGVCIGLFILAIFERYLVAFRRACDAA    76
CnCTR2          -MPACSMNMLWNNQVADTCVVFRSWHISGTWTMILSCLIIIGISVFYSYLLHYIKDYDRH    74
CnCTR3/4        SGHACRISMLLNFNTVDACFLSPNWHIRSKGMFAGSIIGIFFLCVLIELIRRLGREFDRW    92
PdCTR1b         SAARCNMNMLFTWSTQDLCIIFRSWHITGPITLTISLLAIVALVAGFEALRATTARYDAA    72
ScCTR2          GDDTCSMNMLFSWSYKNTCVVFEWWHIKTLPGLILSCLAIFGLAYLYEYLKYCVH-----    107
                      :         .  .                 : :.        :

ScCTR1          WFKKWDKQNKY-------------STLPSANS-------KDEGKHY--------DTE---    209
PdCTR1a         WLDCEMHRRYVAV-------AGK------PGLRERVALHKDAKX4---------------    122
CnCTR1          WRRGQVGYVRPCSNGPLVFSSGKSTSLPPPVLFNRRSSTKKEKDVYNPLTPSDYALEX63-    136
CnCTR2          VAAA----IYSST---------------QQGRRDRDGSPA----ETGLIPPIPTAYG---    108
CnCTR3/4        ----AGVNS---------------TCGELS---------------SVAEYGKD---   115
PdCTR1b         LLKR----RDELP---------------HEELAE---------------TT---------    89
ScCTR2          -----------------------------KRQLS---------------QR---------    114

ScCTR1          --NNFEIQG-LPKLPNLLSDIFVP-------SLMDLFHDIIRAFLVFTSTMIIYMLMLAT    259
PdCTR1a         SENGVE------------EEVVVVQRKGEMTSPWRVSVDPLRAVVDTVIAGMGYLLMLAV    174
CnCTR1          KEKAVERGLVHSHLPKAVRRSLDPGREGRWSRPFRLAVDVPRGLLQALQTLIHYLLMLVV    256
CnCTR2          GIEVGAL-------------NRI-GVTRLPLR--LRLIRAGLYAVTVAISFWLMLVA  149
CnCTR3/4        ---GAQGGAV-------------VRVAPRYVPSWP--HQILRGFIYGSQFTAAFFVMLLG    157
PdCTR1b         --------TL-------------LPGQQQSLRDVR--AKVVRSALYGLETFYAFMIMLLF    126
ScCTR2          --------VL-------------LPNRSLTKINQA--DKVSNSILYGLQVGFSFMLMLVF    151
                                                         .. :        : :**

ScCTR1          MSFVLTYVFAVITGLALSEVFFNRCKIAMLKRWDIQREIQKAKSCPGFX98----------    319
PdCTR1a         MTMNVGYFLSVLAGVFLGSLAIGRYTTSY-----------EGH-----------------    206
CnCTR1          MTFNIWWMISVVIGCGVGEMLFGRFGSSH-----------VGH-----------------    288
CnCTR2          MTYNTYLFSSIVVGAFFGHVIYEDEMDVGAVLSGTS---GKGLACH--------------    192
CnCTR3/4        MYFNVIVLIFIFLGQTVGYMLFGRDTCGGGFDFG-----AQGRCC---------------    197
PdCTR1b         MTYNGQVMIAVGIGAFVGHLAFGGATTA-----------TRETACH--------------    161
ScCTR2          MTYNGWLMLAVVCGAIWGNYSWCTSYSPE---ID-----DSSLACH*-------------    189
                *      .  :  *   .
```
