# Supplementary material for: Characterization of a High-Affinity Copper Transporter CTR1a in the White-Nose Syndrome Causing Fungal Pathogen Pseudogymnoascus destructans
Source: J Fungi (Basel). 2024 Oct 21;10(10):729. doi: 10.3390/jof10100729 (PMC11509074; doi:10.3390/jof10100729)
Supplement: Supplementary file 1 [file jof-10-00729-s001.zip › Supplemental File S2 - Information on Plasmids used in this study .pdf]

Supporting Information File S2

Plasmid pAF23 (pCTR1-yeGFP) tCTR1 (addgene plasmid #223171)

Legend

CTR1 Promoter UNDERLINED

GFP

CTR1 Terminator **BOLD**

Plasmid Sequence:

catgtgtatatatgtatacctatgaatgtcagtaagtatgtatacgaacagtatgatactgaagatgacaaggtaatgcatcattctatacg
tgtcattctgaacgaggcgcgctttcctttttctttttgctttttctttttttttctcttgaactcgacggatctatgcggtgtgaaataccgcacaga
tgcgtaaggagaaaataccgcatcaggaaattgtaagcgttaatattttgttaaaattcgcgttaaatttttgttaaatcagctcattttttaac
caataggccgaaatcggcaaaatcccttataaatcaaaagaatagaccgagatagggttgagtgttgttccagtttggaacaagagtc
cactattaaagaacgtggactccaacgtcaaagggcgaaaaaccgtctatcagggcgatggcccactacgtgaaccatcaccctaa
tcaagttttttggggtcgaggtgccgtaaagcactaaatcggaaccctaaagggagcccccgatttagagcttgacggggaaagccg
gcgaacgtggcgagaaaggaagggaagaaagcgaaaggagcgggcgctagggcgctggcaagtgtagcggtcacgctgcgc
gtaaccaccacacccgccgcgcttaatgcgccgctacagggcgcgtccattcgccattcaggctgcgcaactgttgggaagggcgat
cggtgcgggcctcttcgctattacgccagctggcgaaagggggatgtgctgcaaggcgattaagttgggtaacgccagggttttccca
gtcacgacgttgtaaaacgacggccagtgaattgtaatacgactcactatagggcgaattggagctccaccgcggtggcggccgctc
tagaactagtcacagacattaacccacagtacagacactgcgacaacgtggcaattcgtcgcaatacaacgacacaagtccgattg
ttcctcttcaggagcttcctgaaccaaactttttccgcaaggccgcattttgaaccgtattttgctcgttccagcctttccacgtttttgttatcta
agcaacttggcacatttccctactatactacaaaccgatacgtaaatacttccctaaatagcatatgaattattcagtaatttttaaggatcg
aaactgcacctcaactattcgttactgtggttatgttctcatgtattgatgcaaatcatgggatatttgctcaagacgacggtaaaatgagc
aaaaatggcacgatcctgaaaagagcacttttcaagattcgggctacaaaatgcaacataaaaaatgttgtattgtcatctcgagagg
gtcttgtatgttttattcctcttatgattagttcacattagtaaaacagatacgcagtgtgctcttaataaacaactactccatagctttatttgca
taacaaaacttttaagcacaaacttaaacaggtggagtaatagttcggcggcgactcaaattacatttgttggaagaatcgaatagaa
aataaaaaaaaagtgtattatatttgacattcaaatatgaaaggtgaagaattattcactggtgttgtcccaattttggttgaattagatggtg
atgttaatggtcacaaattttctgtctccggtgaaggtgaaggtgatgctacttacggtaaattgaccttaaaatttatttgtactactggtaa
attgccagttccatggccaaccttagtcactactttcggttatggtgttcaatgttttgcgagatacccagatcacatgaaacaacatgactt
tttcaagtctgccatgccagaaggttatgttcaagaaagaactatttttttcaaagatgacggtaactacaagaccagagctgaagtcaa
gtttgaaggtgataccttagttaatagaatcgaattaaaaggtattgattttaaagaagatggtaacattttaggtcacaaattggaataca
actataactctcacaatgtttacatcatggctgacaaacaaaagaatggtatcaaagttaacttcaaaattagacacaacattgaagat
ggttctgttcaattagctgaccattatcaacaaaatactccaattggtgatggtccagtcttgttaccagacaaccattacttatccactcaa
tctgccttatccaaagatccaaacgaaaagacagaccacatggtcttgttagaatttgttactgctgctggtattacccatggtatggatg
aattgtacaaaggatc**ctaataactagacaaggttcgtccacctatatcttcttcttccaatatctctatacatcaaaataagaata**
tcgttcatttctcagaagctcaaaaaaaatctcaaaatggatacttctaatgccctcatgaagaggaaagaaaataatagaca
cagcgaccttcctataaataacagacttgtatatagttgaatgtctaagtaattttaattcaaaaatccttaactatattaccacctt
gcaataataagagtgaagtgttaaaaatgatacttgcttgccttcatcgagttaccgttgtctcccacgtttctttttctcgttgga
cagaattgcttccgcgcgaacattttccacttttatttgaaagaggacattattcagatcgtgtcagtctcatctaattggcaaag
tggcaattttcgaaataacaagataactgcataaagtaacacttgtcgaattgaaagtattttgccagtgatatttaggttcgagt
aaagaaaatttcataaagaaatcaacaagacacaatgctggaaatctgctcgtcagtggtgctcagaattcgatatcaagcttatc
gataccgtcgacctcgagggggggcccggtaccagcttttgttccctttagtgagggttaatttcgagcttggcgtaatcatggtcatagct
gtttcctgtgtgaaattgttatccgctcacaattccacacaacatacgagccggaagcataaagtgtaaagcctggggtgcctaatgagt
gagctaactcacattaattgcgttgcgctcactgcccgctttccagtcgggaaacctgtcgtgccagctgcattaatgaatcggccaacg
cgcggggagaggcggtttgcgtattgggcgctcttccgcttcctcgctcactgactcgctgcgctcggtcgttcggctgcggcgagcggt
atcagctcactcaaaggcggtaatacggttatccacagaatcaggggataacgcaggaaagaacatgtgagcaaaaggccagca
aaaggccaggaaccgtaaaaaggccgcgttgctggcgtttttccataggctccgcccccctgacgagcatcacaaaaatcgacgct
caagtcagaggtggcgaaacccgacaggactataaagataccaggcgtttccccctggaagctccctcgtgcgctctcctgttccga
ccctgccgcttaccggatacctgtccgcctttctcccttcgggaagcgtggcgctttctcatagctcacgctgtaggtatctcagttcggtgt
aggtcgttcgctccaagctgggctgtgtgcacgaaccccccgttcagcccgaccgctgcgccttatccggtaactatcgtcttgagtcca
acccggtaagacacgacttatcgccactggcagcagccactggtaacaggattagcagagcgaggtatgtaggcggtgctacaga

gttcttgaagtggtggcctaactacggctacactagaagaacagtatttggtatctgcgctctgctgaagccagttaccttcggaaaaag
agttggtagctcttgatccggcaaacaaaccaccgctggtagcggtggtttttttgtttgcaagcagcagattacgcgcagaaaaaaag
gatctcaagaagatcctttgatcttttctacggggtctgacgctcagtggaacgaaaactcacgttaagggattttggtcatgagattatca
aaaaggatcttcacctagatccttttaaattaaaaatgaagttttaaatcaatctaaagtatatatgagtaaacttggtctgacagttacca
atgcttaatcagtgaggcacctatctcagcgatctgtctatttcgttcatccatagttgcctgactccccgtcgtgtagataactacgatacg
ggagggcttaccatctggccccagtgctgcaatgataccgcgagacccacgctcaccggctccagatttatcagcaataaaccagcc
agccggaagggccgagcgcagaagtggtcctgcaactttatccgcctccatccagtctattaattgttgccgggaagctagagtaagt
agttcgccagttaatagtttgcgcaacgttgttgccattgctacaggcatcgtggtgtcacgctcgtcgtttggtatggcttcattcagctccg
gttcccaacgatcaaggcgagttacatgatcccccatgttgtgcaaaaaagcggttagctccttcggtcctccgatcgttgtcagaagta
agttggccgcagtgttatcactcatggttatggcagcactgcataattctcttactgtcatgccatccgtaagatgcttttctgtgactggtga
gtactcaaccaagtcattctgagaatagtgtatgcggcgaccgagttgctcttgcccggcgtcaatacgggataataccgcgccacat
agcagaactttaaaagtgctcatcattggaaaacgttcttcggggcgaaaactctcaaggatcttaccgctgttgagatccagttcgatg
taacccactcgtgcacccaactgatcttcagcatcttttactttcaccagcgtttctgggtgagcaaaaacaggaaggcaaaatgccgc
aaaaaagggaataagggcgacacggaaatgttgaatactcatactcttcctttttcaatattattgaagcatttatcagggttattgtctcat
gagcggatacatatttgaatgtatttagaaaaataaacaaataggggttccgcgcacatttccccgaaaagtgccacctgacggatcg
cttgcctgtaacttacacgcgcctcgtatcttttaatgatggaataatttgggaatttactctgtgtttatttatttttatgttttgtatttggattttaga
aagtaaataaagaaggtagaagagttacggaatgaagaaaaaaaaataaacaaaggtttaaaaaatttcaacaaaaagcgtactt
tacatatatatttattagacaagaaaagcagattaaatagatatacattcgattaacgataagtaaaatgtaaaatcacaggattttcgtg
tgtggtcttctacacagacaagatgaaacaattcggcattaatacctgagagcaggaagagcaagataaaaggtagtatttgttggcg
atccccctagagtcttttacatcttcggaaaacaaaaactatttttctttaatttctttttttactttctatttttaatttatatatttatattaaaaaattt
aaattataattatttttatagcacgtgatgaaaaggaccctaagaaaccattattatcatgacattaacctataaaaataggcgtatcacg
aggccctttcgtctcgcgcgtttcggtgatgacggtgaaaacctctgacacatgcagctcccggagacggtcacagcttgtctgtaagc
ggatgccgggagcagacaagcccgtcagggcgcgtcagcgggtgttggcgggtgtcggggctggcttaactatgcggcatcagag
cagattgtactgagagtgcaccataaattcccgttttaagagcttggtgagcgctaggagtcactgccaggtatcgtttgaacacggcat
tagtcagggaagtcataacacagtcctttcccgcaattttctttttctattactcttggcctcctctagtacactctatatttttttatgcctcggtaa
tgattttcattttttttttccacctagcggatgactcttttttttttcttagcgattggcattatcacataatgaattatacattatataaagtaatgtgat
ttcttcgaagaatatactaaaaaatgagcaggcaagataaacgaaggcaaagatgacagagcagaaagccctagtaaagcgtatt
acaaatgaaaccaagattcagattgcgatctctttaaagggtggtcccctagcgatagagcactcgatcttcccagaaaaagaggca
gaagcagtagcagaacaggccacacaatcgcaagtgattaacgtccacacaggtatagggtttctggaccatatgatacatgctctg
gccaagcattccggctggtcgctaatcgttgagtgcattggtgacttacacatagacgaccatcacaccactgaagactgcgggattg
ctctcggtcaagcttttaaagaggccctaggggccgtgcgtggagtaaaaaggtttggatcaggatttgcgcctttggatgaggcacttt
ccagagcggtggtagatctttcgaacaggccgtacgcagttgtcgaacttggtttgcaaagggagaaagtaggagatctctcttgcga
gatgatcccgcatttcttgaaagctttgcagaggctagcagaattaccctccacgttgattgtctgcgaggcaagaatgatcatcaccgt
agtgagagtgcgttcaaggctcttgcggttgccataagagaagccacctcgcccaatggtaccaacgatgttccctccaccaaaggtg
ttcttatgtagtgacaccgattatttaaagctgcagcatacgatatatata

Plasmid pAF24 (pCTR1-VC83_00191-yeGFP) tCTR1 (Addgene plasmid 2223172)

Legend

CTR1 Promoter UNDERLINED
VC83_00191
Dual Streptag linker
GFP
CTR1 Terminator **BOLD**

Plasmid Sequence:

catgtgtatatatgtatacctatgaatgtcagtaagtatgtatacgaacagtatgatactgaagatgacaaggtaatgcatcattctatacg
tgtcattctgaacgaggcgcgctttcctttttcttttgcttttctttttttttctcttgaactcgacggatctatgcggtgtgaaataccgcacaga
tgcgtaaggagaaaataccgcatcaggaaattgtaagcgttaatattttgttaaaattcgcgttaaatttttgttaaatcagctcattttttaac
caataggccgaaatcggcaaaatcccttataaatcaaaagaatagaccgagatagggttgagtgttgttccagtttggaacaagagtc
cactattaaagaacgtggactccaacgtcaaagggcgaaaaaccgtctatcagggcgatggcccactacgtgaaccatcaccctaa
tcaagttttttggggtcgaggtgccgtaaagcactaaatcggaaccctaaagggagcccccgatttagagcttgacggggaaagccg
gcgaacgtggcgagaaaggaagggaagaaagcgaaaggagcgggcgctagggcgctggcaagtgtagcggtcacgctgcgc
gtaaccaccacacccgccgcgcttaatgcgccgctacagggcgcgtccattcgccattcaggctgcgcaactgttgggaagggcgat
cggtgcgggcctcttcgctattacgccagctggcgaaagggggatgtgctgcaaggcgattaagttgggtaacgccagggttttccca
gtcacgacgttgtaaaacgacggccagtgaattgtaatacgactcactatagggcgaattggagctccaccgcggtggcggccgctc
tagaactagtcacagacattaacccacagtacagacactgcgacaacgtggcaattcgtcgcaatacaacgacacaagtccgattg
ttcctcttcaggagcttcctgaaccaaactttttccgcaaggccgcattttgaaccgtattttgctcgttccagcctttccacgtttttgttatcta
agcaacttggcacatttccctactatactacaaaccgatacgtaaatacttccctaaatagcatatgaattattcagtaattttttaaggatcg
aaactgcacctcaactattcgttactgtggttatgttctcatgtattgatgcaaatcatgggatatttgctcaagacgacggtaaaatgagc
aaaaatggcacgatcctgaaaagagcacttttcaagattcgggctacaaaatgcaacataaaaaatgttgtattgtcatctcgagagg
gtcttgtatgttttattcctcttatgattagttcacattagtaaaacagatacgcagtgtgctcttaataaacaactactccatagctttatttgca
taacaaaacttttaagcacaaacttaaacaggtggagtaatagttcggcggcgactcaaattacatttgttggaagaatcgaatagaa
aataaaaaaaagtgtattatatttgacattcaaatatggctgataatccattcgcaacatcaacgcctacttctggtgatgatatgtctggc
atggatatgtctcatggttcatcacacggcagttctcatgggtcatcgtccggcatgtctatggtcatgacctttcagaataatcctagcac
gcctttgttctctacagcgtggactcctaccggtacggggtcgtatgctgggacttgcattttttaatcgtcttcgctgttctgtttagggttttatt
ggctcttaaagcgcgtcaagaagcaagatggcttgattgcgaaatgcatagaagatacgtggcggtcgctggcaagccaggcttaa
gagaacgtgttgcactacacaaggacgcgaaagcagtcgttttatctgaaaatggcgttgaggaagaggtagtggtggttcaaagaa
agggagaaatgacctcgccatggagggtttccgttgatcctcttagagcagtggtagatactgtgatagctgggatgggttacttgttaat
gcttgcggttatgactatgaacgtggggtacttcctttcagtcctagcaggagtgttcttgggtagtttagccattggtagatacactacttca
tacgaaggacacggtgggtccggcggttcatggtcacacccccaaccagaaaaaggtggtggatcaggagggtcttggagtcacc
ctcaacctgaaaagggaggatcctcgaaaggtgaagaattattcactggtgttgtcccaattttggttgaattagatggtgatgttaatggt
cacaaattttctgtctccggtgaaggtgaaggtgatgctacttacggtaaattgaccttaaaatttatttgtactactggtaaattgccagttc
catggccaaccttagtcactactttcggttatggtgttcaatgttttgcgagatacccagatcacatgaaacaacatgactttttcaagtctg
ccatgccagaaggttatgttcaagaaagaactatttttttcaaagatgacggtaactacaagaccagagctgaagtcaagtttgaaggt
gataccttagttaatagaatcgaattaaaaggtattgattttaaagaagatggtaacattttaggtcacaaattggaatacaactataactc
tcacaatgtttacatcatggctgacaaacaaaagaatggtatcaaagttaacttcaaaattagacacaacattgaagatggttctgttca
attagctgaccattatcaacaaaatactccaattggtgatggtccagtcttgttaccagacaaccattacttatccactcaatctgccttatc
caaagatccaaacgaaaagacagaccacatggtcttgttagaatttgttactgctgctggtattacccatggtatggatgaattgtacaa
aggatc**ctaataactagacaaggttcgtccacctatatcttcttcttccaatatctctatacatcaaaataagaatatcgttcatttc**
tcagaagctcaaaaaaaatctcaaaatggatacttctaatgccctcatgaagaggaaagaaaataatagacacagcgacct
tcctataaataacagacttgtatatagttgaatgtctaagtaattttaattcaaaaatccttaactatattaccaccttgcaataata
agagtgaagtgttaaaaatgatacttgcttgccttcatcgagttatcgttgtctcccacgtttctttttctcgttggacagaattgc
ttccgcgcgaacattttccacttttatttgaaagaggacattattcagatcgtgtcagtctcatctaattggcaaagtggcaatttt
cgaaataacaagataactgcataaagtaacacttgtcgaattgaaagtattttgccagtgatatttaggttcgagtaaagaaaa
tttcataaagaaatcaacaagacacaatgctggaaatctgctcgtcagtggtgctcagaattcgatatcaagcttatcgataccgtcg

acctcgagggggggcccggtaccagcttttgttccctttagtgagggttaatttcgagcttggcgtaatcatggtcatagctgtttcctgtgtg
aaattgttatccgctcacaattccacacaacatacgagccggaagcataaagtgtaaagcctggggtgcctaatgagtgagctaactc
acattaattgcgttgcgctcactgcccgctttccagtcgggaaacctgtcgtgccagctgcattaatgaatcggccaacgcgcggggag
aggcggtttgcgtattgggcgctcttccgcttcctcgctcactgactcgctgcgctcggtcgttcggctgcggcgagcggtatcagctcact
caaaggcggtaatacggttatccacagaatcaggggataacgcaggaaagaacatgtgagcaaaaggccagcaaaaggccag
gaaccgtaaaaaggccgcgttgctggcgtttttccataggctccgcccccctgacgagcatcacaaaaatcgacgctcaagtcagag
gtggcgaaacccgacaggactataaagataccaggcgtttccccctggaagctccctcgtgcgctctcctgttccgaccctgccgctta
ccggatacctgtccgcctttctcccttcgggaagcgtggcgctttctcatagctcacgctgtaggtatctcagttcggtgtaggtcgttcgct
ccaagctgggctgtgtgcacgaaccccccgttcagcccgaccgctgcgccttatccggtaactatcgtcttgagtccaacccggtaag
acacgacttatcgccactggcagcagccactggtaacaggattagcagagcgaggtatgtaggcggtgctacagagttcttgaagtg
gtggcctaactacggctacactagaagaacagtatttggtatctgcgctctgctgaagccagttaccttcggaaaaagagttggtagctc
ttgatccggcaaacaaaccaccgctggtagcggtggtttttttgtttgcaagcagcagattacgcgcagaaaaaaaggatctcaagaa
gatcctttgatcttttctacggggtctgacgctcagtggaacgaaaactcacgttaagggattttggtcatgagattatcaaaaaggatctt
cacctagatccttttaaattaaaaatgaagttttaaatcaatctaaagtatatatgagtaaacttggtctgacagttaccaatgcttaatcag
tgaggcacctatctcagcgatctgtctatttcgttcatccatagttgcctgactccccgtcgtgtagataactacgatacgggagggcttac
catctggccccagtgctgcaatgataccgcgagacccacgctcaccggctccagatttatcagcaataaaccagccagccggaagg
gccgagcgcagaagtggtcctgcaactttatccgcctccatccagtctattaattgttgccgggaagctagagtaagtagttcgccagtt
aatagtttgcgcaacgttgttgccattgctacaggcatcgtggtgtcacgctcgtcgtttggtatggcttcattcagctccggttcccaacga
tcaaggcgagttacatgatcccccatgttgtgcaaaaaagcggttagctccttcggtcctccgatcgttgtcagaagtaagttggccgca
gtgttatcactcatggttatggcagcactgcataattctcttactgtcatgccatccgtaagatgcttttctgtgactggtgagtactcaacca
agtcattctgagaatagtgtatgcggcgaccgagttgctcttgcccggcgtcaatacgggataataccgcgccacatagcagaacttta
aaagtgctcatcattggaaaacgttcttcggggcgaaaactctcaaggatcttaccgctgttgagatccagttcgatgtaacccactcgt
gcacccaactgatcttcagcatcttttactttcaccagcgtttctgggtgagcaaaaacaggaaggcaaaatgccgcaaaaaaggga
ataagggcgacacggaaatgttgaatactcatactcttcctttttcaatattattgaagcatttatcagggttattgtctcatgagcggataca
tatttgaatgtatttagaaaaataaacaaataggggttccgcgcacatttccccgaaaagtgccacctgacggatcgcttgcctgtaactt
acacgcgcctcgtatcttttaatgatggaataatttgggaatttactctgtgtttatttatttttatgttttgtatttggattttagaaagtaaataaa
gaaggtagaagagttacggaatgaagaaaaaaaaaataaacaaaggtttaaaaaatttcaacaaaaagcgtactttacatatatattt
attagacaagaaaagcagattaaatagatatacattcgattaacgataagtaaaatgtaaaatcacaggattttcgtgtgtggtcttctac
acagacaagatgaaacaattcggcattaatacctgagagcaggaagagcaagataaaaggtagtatttgttggcgatccccctaga
gtcttttacatcttcggaaaacaaaaactatttttttctttaatttctttttttactttctatttttaatttatatatttatattaaaaaatttaaattataattat
ttttatagcacgtgatgaaaaggaccctaagaaaccattattatcatgacattaacctataaaaataggcgtatcacgaggccctttcgt
ctcgcgcgtttcggtgatgacggtgaaaacctctgacacatgcagctcccggagacggtcacagcttgtctgtaagcggatgccggg
agcagacaagcccgtcagggcgcgtcagcgggtgttggcgggtgtcggggctggcttaactatgcggcatcagagcagattgtactg
agagtgcaccataaattcccgttttaagagcttggtgagcgctaggagtcactgccaggtatcgtttgaacacggcattagtcagggaa
gtcataacacagtcctttcccgcaattttctttttctattactcttggcctcctctagtacactctatatttttttatgcctcggtaatgattttcattttttt
tttccacctagcggatgactcttttttttttcttagcgattggcattatcacataatgaattatacattatataaagtaatgtgatttcttcgaagaat
atactaaaaaatgagcaggcaagataaacgaaggcaaagatgacagagcagaaagccctagtaaagcgtattacaaatgaaac
caagattcagattgcgatctctttaaagggtggtcccctagcgatagagcactcgatcttcccagaaaaagaggcagaagcagtagc
agaacaggccacacaatcgcaagtgattaacgtccacacaggtatagggtttctggaccatatgatacatgctctggccaagcattcc
ggctggtcgctaatcgttgagtgcattggtgacttacacatagacgaccatcacaccactgaagactgcgggattgctctcggtcaagc
ttttaaagaggccctaggggccgtgcgtggagtaaaaaggtttggatcaggatttgcgcctttggatgaggcactttccagagcggtggt
agatctttcgaacaggccgtacgcagttgtcgaacttggtttgcaaagggagaaagtaggagatctctcttgcgagatgatcccgcattt
tcttgaaagctttgcagaggctagcagaattaccctccacgttgattgtctgcgaggcaagaatgatcatcaccgtagtgagagtgcgtt
caaggctcttgcggttgccataagagaagccacctcgcccaatggtaccaacgatgttccctccaccaaaggtgttcttatgtagtgac
accgattatttaaagctgcagcatacgatatatata

Recombinant protein expressed by pAF24

Legend

- *Pd*CTR1a (VC83_00191)
- Strep-tag II Linker
- yeGFP

Protein Sequence:

MADNPFATSTPTSGDDMSGMDMSHGSSHGSSHGSSSGMSMVMTFQNNPSTPLFSTAWTPT
GTGSYAGTCIFLIVFAVLFRVLLALKARQEARWLDCEMHRRYVAVAGKPGLRERVALHKDAKAV
VLSENGVEEEVVVVQRKGEMTSPWRVSVDPLRAVVDTVIAGMGYLLMLAVMTMNVGYFLSVL
AGVFLGSLAIGRYTTSYEGHGGSGGSWSHPQPEKGGGSGGSWSHPQPEKGGSSKGEELFT
GVVPILVELDGDVNGHKFSVSGEGEGDATYGKLTLKFICTTGKLPVPWPTLVTTFGYGVQCFAR
YPDHMKQHDFFKSAMPEGYVQERTIFFKDDGNYKTRAEVKFEGDTLVNRIELKGIDFKEDGNIL
GHKLEYNYNSHNVYIMADKQKNGIKVNFKIRHNIEDGSVQLADHYQQNTPIGDGPVLLPDNHYL
STQSALSKDPNEKTDHMVLLEFVTAAGITHGMDELYKGS*
