# Supplementary material for: Characterization of a High-Affinity Copper Transporter CTR1a in the White-Nose Syndrome Causing Fungal Pathogen Pseudogymnoascus destructans
Source: J Fungi (Basel). 2024 Oct 21;10(10):729. doi: 10.3390/jof10100729 (PMC11509074; doi:10.3390/jof10100729)
Supplement: Supplementary file 1 [file jof-10-00729-s001.zip › Supplemental File S3 - Flow cytometry histograms .pdf]

Supplemental File S3. Flow cytometry histograms of recombinant yeast strains used in this study cultured under Cu-stress conditions.

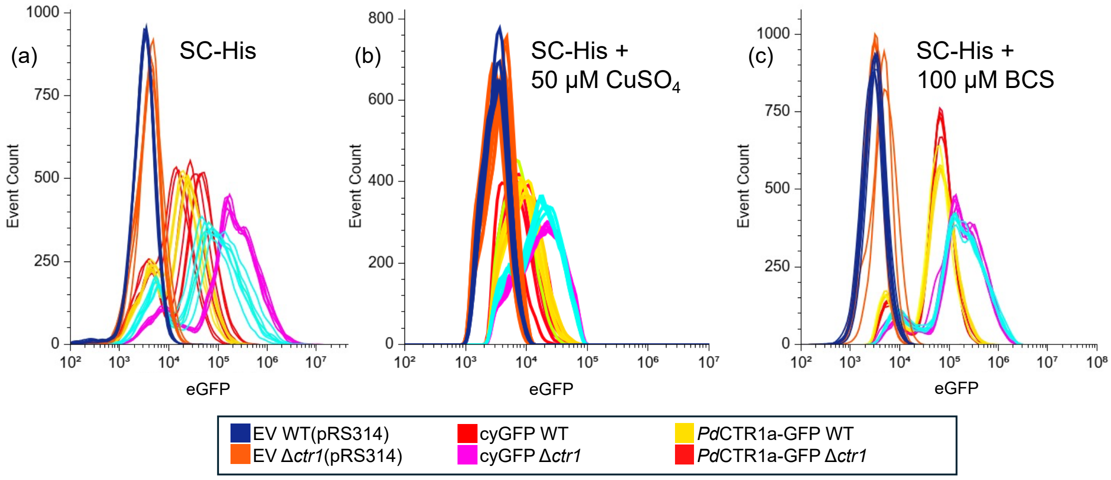

Supplemental Figure S1. Flow cytometry of recombinant *Saccharomyces cerevisiae* strains. GFP fluorescence profiles of BY4741 (WT) and BY4741*ctr1*Δ (*ctr1*Δ) yeast harboring empty vector (EV), cytosolic-GFP (CyGFP) or *Pd*CTR1A-GFP under the control of the *S. cerevisiae* Ctr1 promoter and terminator. Yeast was grown to the log phase in SC-His under varying Cu-stress conditions: (a) control (i.e., SC-His), (**b**) SC-His + 50 µM Cu-sulfate, (**c**) SC-His + 100 µM BCS. A total of 10,000 events are displayed per trace. Traces are normalized to the background fluorescence of yeast harboring the pRS313 empty vector plasmid alone set to an intensity of ~1 x 10^3 eGFP fluorescent units.

Supplemental Table S1. Calculated mean fluorescence intensity for flow cytometry histograms.

SC-His	Mean fluorescence Intensity – Biological Replicate						
Strain	1	2	3	4	5	6	Average
EV WT(pRS314)	3598	3585	3668	3442	3770	3812	3646
EV Δ*ctr1*(pRS314)	4385	4371	5426	4492	4727	4771	4695
cyGFP WT	167918	225250	137275	129137	92500	149379	150243
cyGFP Δ*ctr1*	310911	332913	359043	379391	354896	349508	347777
*Pd*CTR1a-GFP WT	30504	31112	22027	24166	28817	29853	44285
*Pd*CTR1a-GFP Δ*ctr1*	17195	15387	27756	47230	35174	44285	31171

SC-His + Cu-sulfate	Biological Replicate						
Strain	1	2	3	4	5	6	Average
EV WT(pRS314)	5698	4227	6031	8789	8120	6980	6641
EV Δ*ctr1*(pRS314)	4022	4927	4088	4243	4658	5052	4498
cyGFP WT	29702	28891	28381	27851	32162	30671	29610
cyGFP Δ*ctr1*	38745	40626	38583	40402	37055	41645	39509
*Pd*CTR1a-GFP WT	10781	9803	12953	10420	14011	13440	11901
*Pd*CTR1a-GFP Δ*ctr1*	9282	8780	12524	8130	5374	9091	8864

SC-His + BCS	Biological Replicate						
Strain	1	2	3	4	5	6	Average
EV WT(pRS314)	3852	3998	5183	3336	3233	3122	3787
EV Δ*ctr1*(pRS314)	6387	3681	3227	3192	3460	4718	4111
cyGFP WT	347639	299445	306112	285643	292118	296681	304606
cyGFP Δ*ctr1*	243429	320061	330413	259905	266619	266653	281180
*Pd*CTR1a-GFP WT	64642	83638	75596	75823	79546	76814	76010
*Pd*CTR1a-GFP Δ*ctr1*	71445	72198	73974	70529	72940	76279	72894
