# Supplementary material for: Characterization of a High-Affinity Copper Transporter CTR1a in the White-Nose Syndrome Causing Fungal Pathogen Pseudogymnoascus destructans
Source: J Fungi (Basel). 2024 Oct 21;10(10):729. doi: 10.3390/jof10100729 (PMC11509074; doi:10.3390/jof10100729)
Supplement: Supplementary file 1 [file jof-10-00729-s001.zip › Table S2 Primers.pdf]

SI Table S1. Table of primers used in this study.

Component	Template	Primers	Primer Sequence
CTR Promoter – GFP terminator cassette	Pytk110-DN6	oRLP497	cataggtctcactagtcacagacattaacccacagtacagacac
		oRLP498	ctaacggtctcgaattctgagcaccactgacgagcagatt
RT-qPCR	Actin cDNA	oRLP478	ATCACACCTTCTACAACGAGC
		oRLP479	GGCGTTGAAAGTCTCGAAAAC
RT-qPCR	*Pd*Ctr1a cDNA	oRLP198	GACAAGTCCGTGGAGAGTTAG
		oRLP199	AAGCTACCTAGGAAAACGCC
RT-qPCR	*Pd*Ctr1b cDNA	oRLP200	CCCCATAACACTCACCATCTC
		oRLP201	GTCTCAGCAAGTTCCTCGTG
